# Recent advances in cytokinesis: understanding the molecular underpinnings

**DOI:** 10.12688/f1000research.16502.1

**Published:** 2018-11-26

**Authors:** Yinan Liu, Douglas Robinson

**Affiliations:** 1 Departments of Cell Biology and Pharmacology and Molecular Sciences, Johns Hopkins University School of Medicine, Baltimore, Maryland, 21205, USA

**Keywords:** cytokinesis, myosin, NoCut, ESCRTIII, FtsZ

## Abstract

During cytokinesis, the cell employs various molecular machineries to separate into two daughters. Many signaling pathways are required to ensure temporal and spatial coordination of the molecular and mechanical events. Cells can also coordinate division with neighboring cells to maintain tissue integrity and flexibility. In this review, we focus on recent advances in the understanding of the molecular underpinnings of cytokinesis.

## Introduction

Cytokinesis requires remodeling of the cell cortex, constriction of the cleavage furrow, and finally severing of the plasma membrane. Many molecular and mechanical mechanisms are highly conserved across the many kingdoms of life and have been well studied in eukaryotic models, including yeast, amoebas, worms, flies, and mammalian cells, and in prokaryotic models ^1, 2^. In this review, we focus on a few eukaryotic molecular mechanisms that have received extensive interest during the past few years. Microtubules and actomyosin networks assemble the contractile machinery, which in many cases is the main driver of cleavage furrow formation ^3^. The endosomal sorting complex required for transport III (ESCRTIII) assists with recruiting membrane components to the midbody and then facilitates plasma membrane abscission ^4^. The Aurora-B-dependent NoCut pathway regulates chromosome separation and clears lagging chromatids from the midbody ^5^. In multicellular scenarios, dividing cells can coordinate with neighboring non-dividing cells through mechanotransduction via cell–cell junctions to ensure tissue integrity and flexibility ^6^.

## Cytokinesis requires microtubule dynamics, an assembly of actomyosin networks, and their regulators

### Microtubules

**Figure 1.**
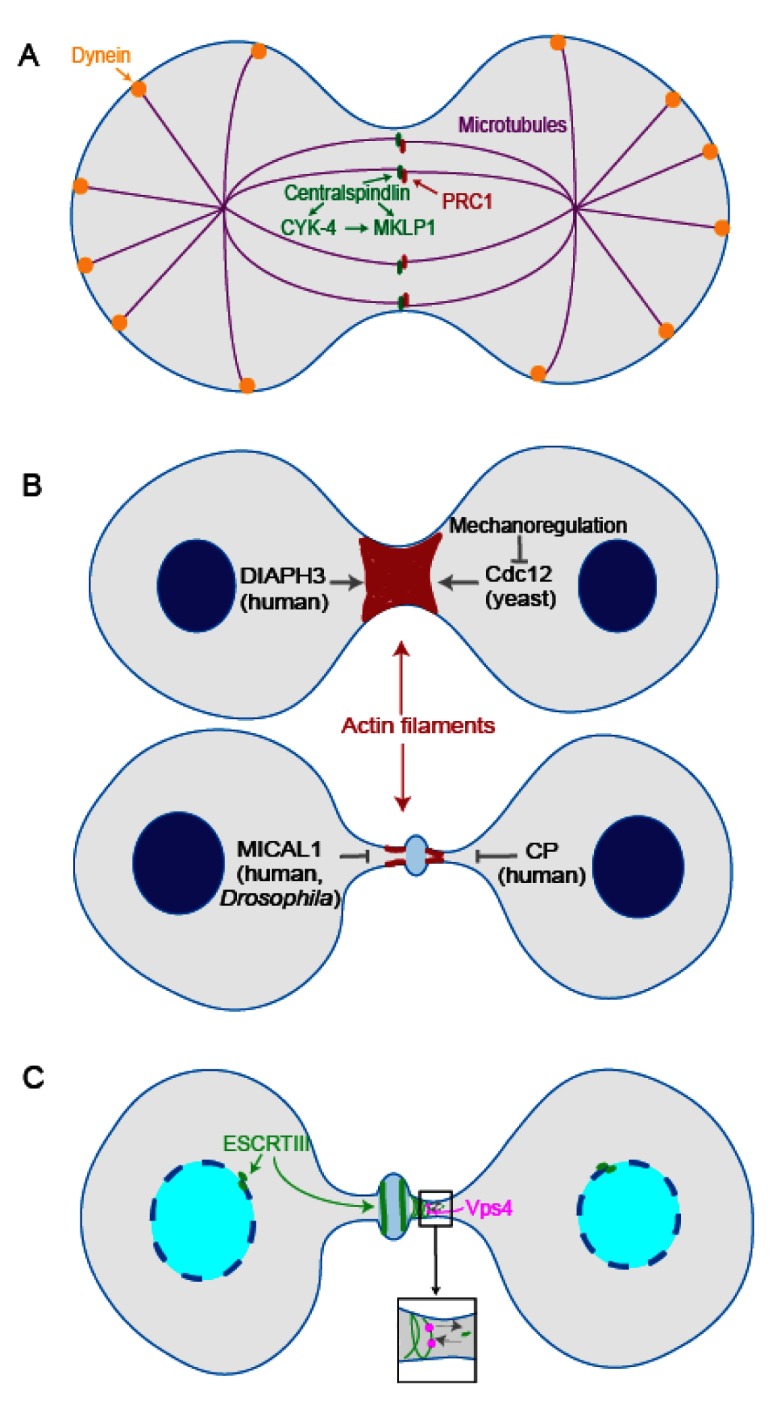
Cytokinesis from spindle maturation to abscission. ( **A**) In *Caenorhabditis elegans*, centralspindlin non-motor subunit CYK-4 binds to the motor subunit MKLP1, activating MKLP1’s antiparallel microtubule bundling activity. Centralspindlin and *C. elegans* orthologues of human PRC1 interact to resist cortical dynein pulling forces and prevent midzone rupture. ( **B**) During actomyosin contraction, DIAPH3 in HeLa cells facilitates *de novo* synthesis of β-actin to ensure furrow ingression. Formin Cdc12 inhibition by mechanoregulation in fission yeast facilitates contractile ring assembly. On the other hand, MICAL1 oxidation of actin in human and *Drosophila* cells leads to depolymerization of F-actin filaments at the intercellular bridge. Actin capping protein in HeLa cells antagonizes actin polymerization to facilitate progression to abscission. ( **C**) In HeLa cells, ATPase Vps4 facilitates endosomal sorting complex required for transport III (ESCRTIII)’s turnover, contributing to ESCRTIII filament assembly at the midbody. ESCRTIII also helps in reforming the nuclear envelope during telophase and cytokinesis.

The mitotic spindle plays multiple roles during cell division, including segregation of chromosomes, positioning of the cleavage furrow, and separation of daughter cells ^7^. In addition to kinetochore microtubules, the spindle includes the astral microtubules and the central spindle. Astral microtubules contact the cortex and are pulled on by cortical dynein to help elongate the spindle. The central spindle is composed of two populations of microtubules, each emanating from the opposing poles that form anti-parallel bundles that span the spindle midzone. The central spindle maintains the structure of the mitotic spindle, which is under constant mechanical stress from the pulling forces placed on the astral microtubules. Recently, the mechanism of centralspindlin microtubule bundling and how central spindle structure maintains its integrity under mechanical stress has emerged. The non-motor subunit of centralspindlin in *Caenorhabditis elegans*, CYK-4, binds to the neck domain on MKLP1 and reconfigures MKLP1 dimer so that it can bundle the antiparallel microtubules ^8^. Moreover, the *C. elegans* orthologues of human PRC1 interact directly with centralspindlin. Both PRC1 and centralspindlin are microtubule bundlers, and this interaction resists the cortical pulling forces exerted during cytokinesis to prevent midzone rupture ( Figure 1A) ^9^.

### Actomyosin network

The progression of cytokinesis is coordinated by cortical actomyosin assembly, contraction, and resolution. In amoebozoan and metazoan cells, actin filaments assemble into bundles and/or networks at the cleavage furrow. These networks confer the mechanical features of the cortex and mediate contractility. On the molecular and network scale, a combination of physical features, including myosin II contractility, actin polymer assembly/disassembly dynamics, and actin filament buckling, contribute to network contraction ^10^.

Actin filaments go through dynamic assembly and disassembly during cytokinesis. Actin can be recruited to the cleavage furrow cortex through cortical flow or produced *de novo* at the cleavage furrow ^11, 12^. DIAPH3, a formin, is an actin nucleator that is recruited to the cleavage furrow cortex by Anillin ^13^. In the human cervical carcinoma HeLa cell line, DIAPH3 nucleates only β-actin at the cleavage furrow, and this *de novo* synthesis of β-actin ensures stable furrow ingression ^14^. Interestingly, the fission yeast formin Cdc12 is inhibited by the pulling forces generated by myosin II, which in turn facilitates contractile ring assembly by allowing the actin structures to condense, leading to ring assembly ( Figure 1B) ^15^. As cytokinesis proceeds, eventually the actin filaments have to be cleared from the intercellular bridge to allow abscission to complete ^16^. Although classical activities such as cofilin-mediated actin disassembly contribute to this actin removal, Fremont *et al*. reported that in both human and *Drosophila* cells, the oxidation of actin by MICAL1, a redox enzyme, can also induce depolymerization of F-actin filaments at the intercellular bridge ^17^. In HeLa cells, actin capping protein, a barbed-end actin-binding protein complex, also counteracts formin-dependent F-actin polymerization to modulate actin filament formation as furrow ingression proceeds towards abscission ( Figure 1B) ^18^. In fission yeast, the mechanisms of actin filament disassembly during ring constriction remain unclear ^19^, but one possibility is that the actomyosin bundles are expelled from the contractile ring as the cleavage furrow cortex curvature increases ^20^. Thus, actin dynamics are achieved through a combination of biochemical and mechanical events.

The motor protein myosin works cooperatively with actin to accomplish constriction, yet how myosin II is organized at the contractile ring is not thoroughly understood. Moreover, it is not completely clear whether a specific structural organization is even important for many types of cells to divide or whether some of the patterns observed are effects rather than causes of cleavage furrow contractility. Nevertheless, Wollrab *et al*. described that myosin motors organize into clusters in both mammalian cells and fission yeast, but each have very distinct dynamics. The mammalian myosin II clusters remain relatively still while the yeast myosin II clusters rotate. These differences in the cluster dynamics between the species may indicate specific roles for myosin clusters during cytokinesis ^21^. Alternatively, this may reflect the unique mechanical scenarios in which these clusters exist. Apart from the organization of each myosin type, different isoforms of myosin provide specific functions during cytokinesis, exemplified in fission yeast. First, the fission yeast myosin II paralogs assemble into structures that appear to be unique when compared to conventional bipolar filaments observed in most other systems ^22, 23^. Second, Myo2 is responsible for the assembly and constriction of actin, but Myp2 and Myo51 provide distinct roles ^24^. Myo2 and Myp2 can complete cytokinesis independently, although Myp2 localizes to a different domain of the contractile ring from Myo2. Furthermore, the myosin V paralog Myo51 arrives at the contractile ring after Myo2 and promotes node interactions ^24^.

Besides the structural and molecular distinctions of myosin, the dynamics of myosin II and its partners (in *Dictyostelium*, IQGAPs and cortexillin; in mammalian cells, Anillin) at the cleavage furrow also affect the progression of cytokinesis. These networks of proteins are highly responsive to mechanical stress, and their recruitment to the cleavage furrow cortex is heavily tuned by these stresses ^25– 27^. This mechanoresponsiveness of the cytokinesis machinery endows the cell with the ability to undergo cell division in diverse mechanical contexts, providing yet another essential mechanism for ensuring genomic fidelity throughout the process.

In addition to conventional contractility (filament sliding by myosin II), myosin II and its actin crosslinking partners provide load-resisting tension and strain stiffening as well as promote cortical tension. The cortical tension component initially resists cell deformation but later in cytokinesis, as the axial ratio (length/diameter) of the furrow increases, leads to Laplace (fluid) pressures that can drive furrow ingression ^28, 29^. These properties in combination ensure robust furrow ingression ^3, 25, 30, 31^. For example, these features readily account for how cells devoid of myosin II function can divide ^28, 29, 32^. The lower-level eukaryote *Giardia lamblia* performs cytokinesis using actin filaments but no myosin II ^31^. Here, the flagella provide force generation to generate the appropriate shapes of dividing cells. It has been proposed that these larger-scale mechanics (cortical tension) combined with the elongated cell geometry lead to Laplace pressure-mediated furrow ingression in a manner like that seen in *myosin II* null cells in higher eukaryotes.

### Rho GTPase regulators

In higher eukaryotic systems, one critical regulatory activity is that of the Rho GTPase family of molecular switches. Rho GTPases, including RhoA, Rac1, and Cdc42, regulate spindle assembly and positioning as well as actomyosin contractile ring formation and activation. The key cytokinesis Rho GTPase is RhoA, which acts on downstream substrates, such as formin and ROCK, to regulate actomyosin contractile machinery. RhoA is itself regulated by several GAPs and GEFs. Notably, Ect2, which is activated by Cdk1, is a RhoA GEF that activates RhoA in coordination with the centralspindlin subunit MgcRacGAP ^33^.

Although regulators of RhoA have been extensively studied, it has been somewhat controversial how MgcRacGAP can promote RhoA activation, since it is counterintuitive that a GAP protein could activate a Rho GTPase. Moreover, previously it was shown that the *C. elegans* MgcRacGap homologue (CYK-4) inactivates Rac to promote cytokinesis and does not work directly on RhoA ^34, 35^. However, MgcRacGAP promotes Ect2 accumulation to centralspindlin, which is required for Ect2 to interact with RhoA ^36^. The GAP activity of MgcRacGAP is also important for restricting active RhoA at the cleavage furrow in epithelial cells ^37^. Surprisingly, another RhoA regulator that has recently been identified is the transcription factor YAP. In cultured mammalian cells, YAP localizes to the central spindle and midbody ring during cytokinesis and also affects RhoA, Ect2, and MgcRacGAP localization ^38^. Besides individual RhoA regulatory mechanisms, interestingly, excitable waves of Rho have been observed leading F-actin waves within the cleavage furrow of starfish zygotes and frog blastomeres. These Rho waves are also regulated by Cdk1 and Ect2 and antagonized by F-actin, and the Rho–actin waves enable robust coordination of the cortex with spindle positioning ^39^. Thus, many levels of RhoA regulation appear to be at work and generally feed through Ect2, MgcRacGAP, and F-actin.

## Cytokinesis completion depends on ESCRTIII-mediated lipid dynamics

Membrane trafficking and remodeling play significant roles at the abscission stage during cytokinesis. ESCRT is responsible for membrane constriction and fission. ESCRT has five subfamilies, including ESCRTIII and AAA ATPase Vps4. It has been proposed that ESCRTIII forms spiral structures on the membrane and provides the constriction force to complete abscission ( Figure 1C). Vps4 is responsible for recycling ESCRTIII components back into the cytoplasm, and the interaction of apoptosis-linked gene 2-interacting protein (ALIX) with ESCRTIII is also essential for cytokinesis completion. The underlying mechanisms of ESCRTIII function during abscission are still being actively studied. On one hand, ESCRTIII long filaments appear to help facilitate constriction. On the other hand, ESCRTIII generates vesicles at the midbody, and recycling endosome accumulation at the midbody appears to be required for abscission ^4, 40, 41^.

ESCRTIII filaments are highly dynamic, and midbody ESCRTIII subunits continuously exchange with subunits from the cytoplasm. In HeLa cells, ATPase Vps4 is responsible for ESCRTIII’s dynamic turnover and contributes to ESCRTIII filament growth ( Figure 1C). This dynamic turnover behavior might help ESCRTIII adapt to different membrane curvatures ^42^. Working upstream of ESCRTIII assembly at the abscission site, ALIX binds to the membrane remodeling machinery and the ESCRTIII subunit charged multi-vesicular body protein 4 (CHMP4), thus facilitating the assembly of ESCRTIII filaments. The timing of ALIX recruitment and activity is regulated by phosphorylation. Specifically, in *Xenopus laevis* oocytes, ALIX is phosphorylated at M phase, which leads to the opening up of the ALIX conformation and the subsequent recruitment of CHMP4 to the midbody ^43^. In addition, ESCRTIII helps in reforming the nuclear envelope during telophase and cytokinesis. Both Stenmark’s lab and Carlton’s lab showed that in HeLa cells ESCRTIII localizes to the sites where nuclear envelope repair is occurring, helping to reseal the nuclear envelope ( Figure 1C) ^44, 45^. Vps4 localizes around the chromatin disk and assists ESCRTIII nuclear envelope sealing ^44^. Thus, ESCRTIII plays multiple critical roles in sealing the membrane, both at the nuclear envelope and at the intercellular bridge, leading to final cell separation.

## Cytokinesis abscission checkpoint and cellular shape adaptation prevent chromatin bridge breakage

During mitosis, chromosomes go through dynamic topological changes as well as spatial redistribution. Chromosomes are condensed during prophase, connected to spindle microtubules during prometaphase, and aligned at the spindle equator during metaphase. The sister chromatids are then separated during anaphase. During telophase, the nuclear envelope reforms, and the chromosomes are decondensed. The subsequent cytokinesis ensures separation of cytoplasm and the two daughter nuclei ^46^. However, unresolved DNA structures formed during replication or recombination, defects in condensation, aberrant chromosome attachment to the spindle microtubules, or sister chromatid separation error lead to lagging chromosomes that remain at the cell equator during cytokinesis. The presence of lagging chromosomes can then lead to unwanted DNA damage, resulting in aneuploidy or polyploidy caused by regression of cytokinesis ^47^. Structurally, the presence of lagging chromosomes leads to the formation of anaphase bridges as the cleavage furrow approaches completion. The Aurora B-dependent NoCut checkpoint pathway (NoCut) is charged with helping to resolve these anaphase bridges ^5^. This pathway is aided by the actomyosin network, which maintains the intercellular bridge opening, helping the chromosomes to segregate before abscission and preventing errors from forming in the first place ^48, 49^.

**Figure 2.**
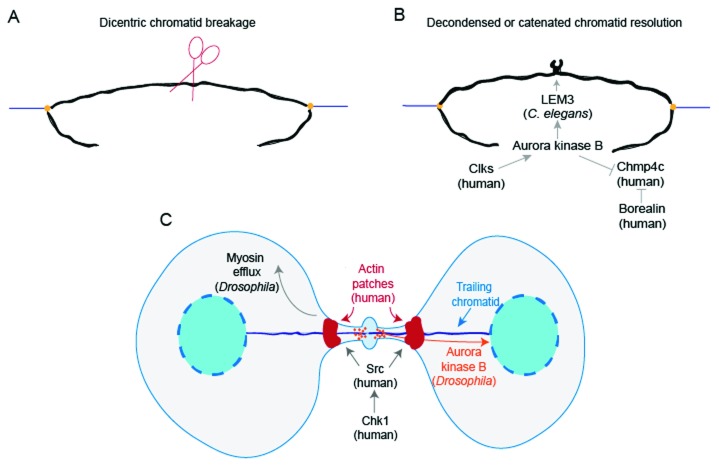
Resolution of chromatin bridges. ( **A**) A trailing dicentric chromatid in *Saccharomyces cerevisiae* is cleaved during cytokinesis. ( **B**) A chromatin bridge caused by decondensed or catenated chromatin is resolved by the Aurora B kinase-dependent NoCut pathway. In *Caenorhabditis elegans*, Aurora B facilitates nuclease LEM-3 recruitment to the midbody to help resolve chromatin bridges. In human cells, Clks phosphorylate Aurora B kinase to activate the NoCut checkpoint. Specifically, in HeLa cells, Aurora B phosphorylation of charged multi-vesicular body protein 4C (CHMP4C) interferes with its membrane remodeling activity. Borealin also inhibits CHMP4C association with the membrane. ( **C**) In human cells, Chk1 activates Src to form actin patches at the base of chromatin bridges, and Chk1 and Src work cooperatively with Aurora kinase B to delay abscission. In *Drosophila* neuronal stem cells, myosin II efflux from the ring to the poles facilitates extra cell elongation to allow for the clearance of trailing chromatids. Moreover, in *Drosophila* larval neuroblasts, Aurora B kinase helps inclusion of the trailing chromatid into the reforming nuclei.

Until recently, it was unclear which specific types of chromosomal structural challenges or attachment errors NoCut resolves. It was elucidated recently that NoCut appears to delay abscission after DNA damage caused by replication stress ^50^. Specifically, Aurora B inhibits abscission only when chromatin bridges are caused by decondensed or catenated chromatin but not when chromatin bridges are caused by dicentric chromatin ( Figure 2). On the other hand, dicentric chromosomes positioned at the midbody during cytokinesis can also create chromatin bridges, but the physical mechanism behind the breakage of dicentric chromatin has been unknown ^51^. Lopez *et al.* argued that instead of being sheared or resolved by a nuclease during anaphase, dicentric chromatins are cleaved during cytokinesis, at least in budding yeast *Saccharomyces cerevisiae* ( Figure 2A) ^52^. They discovered that dicentrics without telomere fusion were prone to breakage at pericentromeric regions, and this breakage required actomyosin network activity during cytokinesis.

Although the NoCut checkpoint prevents unresolved chromatin breakage, how NoCut operates at the chromatin level to resolve chromatin bridges, allowing abscission, has not been well elucidated. LEM-3, a mitotic nuclease, localizes to the midbody in an AIR-2/Aurora B kinase-dependent manner and assists in resolving chromatin bridges in *C. elegans* ( Figure 2B) ^53^.

To turn on the NoCut checkpoint, Aurora kinase B, a subunit of chromosomal passenger complex (CPC), is activated by phosphorylation, which in turn activates downstream substrates, such as CHMP4C ^54, 55^. CHMP4C assembles into long filaments to assist membrane constriction. Petsalaki *et al.* discovered that Cdc-like kinases Clk1, Clk2, and Clk4 in human colon carcinoma BE cells and HeLa cells phosphorylate Aurora kinase B at S33, and subsequently Aurora B phosphorylates CHMP4C. These events prevent late-cytokinesis chromatin breakage ( Figure 2B) ^56^. Moreover, Aurora B phosphorylation of CHMP4C in HeLa cells interferes with its membrane remodeling activity, and Borealin, another subunit of CPC, interacts with CHMP4 and interferes with its association with the membrane ( Figure 2B) ^57, 58^. In combination, the evidence above indicates that CPC and Clks are critical to the regulation of the NoCut checkpoint ^57^.

To prevent lagging chromosomes from being broken or excluded from the nuclei, the shape of the dividing cell is adapted for the clearance of chromatids, and nuclear envelope formation is also coordinated with the inclusion of trailing chromatids ^48, 49, 59^. At the base of chromatin bridges, actin patches form to support the structure of the bridge until chromosomes are resolved ^60^. Actin patch formation is stimulated when the nonreceptor tyrosine kinase Src is activated by Chk1 kinase, and Chk1 and Src work cooperatively with Aurora kinase B to delay abscission in BE and HeLa cells ( Figure 2C) ^48^. Interestingly, in *Drosophila* neuronal stem cells, the cell undergoes a special transient extra elongation just to allow for clearance of the extra-long chromatids from the midbody ^61^. The Rho-GEF (pebble) promotes myosin II transient efflux from the ring to the poles, and this efflux facilitates extra cell elongation ( Figure 2C) ^49^. In addition, Karg *et al.* described that in *Drosophila* larval neuroblasts nuclear envelope formation and the inclusion of trailing acentric chromosomes into the forming nuclei during telophase and cytokinesis require Aurora kinase B activity, adding yet another layer of function to Aurora kinase B ( Figure 2C) ^59^. In summary, the abscission checkpoint and adaptation of cellular shape and organelle structures during cytokinesis cooperate to prevent the lagging chromosomes from being broken, thereby helping to prevent aneuploidy formation. Given the critical importance of genomic fidelity to cell health and organismal viability, it is not surprising that nature has evolved multiple layers of control to ensure cytokinesis completes successfully.

## Cytokinesis at the tissue level reveals coordination of dividing cells with neighboring cells

**Figure 3.**
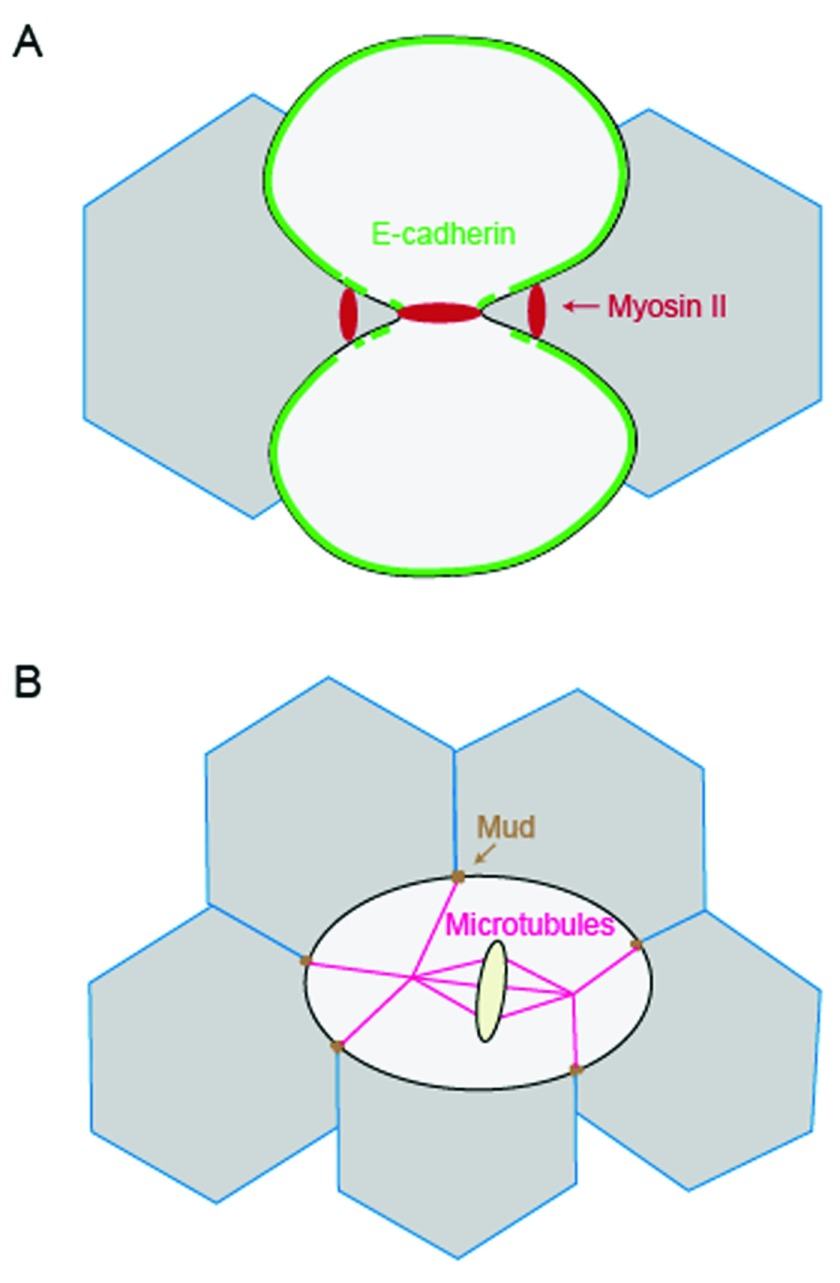
Intercellular junctions guide contractile machinery assembly and division plane specification in multicellular scenarios. ( **A**) In the *Drosophila* notum epithelium, E-cadherin dilution caused by elongation of ingressing adherens junctions induces the accumulation of myosin II at adherens junctions between neighboring cells. ( **B**) In the *Drosophila* pupal notum epithelium, tricellular junctions orient the mitotic spindle via Mud, and the resulting tricellular junction bipolarity facilitates cell mechanical strain-induced polarization, guiding the axis of division.

At the tissue level, the mechanotransduction and regulation of cell polarity between dividing cells and neighboring cells are critical to the integrity and dynamics of epithelial structure ^6^. In two model systems, the *Drosophila* notum epithelium and *Xenopus* embryos, cell–cell junctions are important transducers of mechanosensation and mediators of cell shape regulation. In the *Drosophila* notum epithelium, E-cadherin dilution caused by elongation of ingressing adherens junctions leads to local cortex detachment of neighboring cells. This E-cadherin dilution causes the accumulation of myosin II at adherens junctions of neighboring cells via actomyosin flow ( Figure 3A) ^62^. Thus, this mechanoresponsive pathway linking shape and mechanical properties of dividing cells with those of its neighboring cells helps coordinate epithelial cell dynamics. In contrast, in *X. laevis* embryos, adherens junctions are stabilized at the ingressing furrow between dividing cells and neighboring cells. This stabilization of adherens junctions is proposed to be achieved by the higher tension created by pulling forces at the furrow and leads to the recruitment of vinculin to adherens junctions ^63^. However, the actomyosin-generated tension on cadherin at adherens junctions may not be significantly different between neighboring non-dividing cells and neighboring dividing cells in *Xenopus* embryos ^64^.

Another important question is how connections between neighboring cells regulate the polarity of epithelial cell division, which in turn helps specify patterning in the epithelia. In the *Drosophila* pupal notum epithelium, tricellular junctions serve as landmarks to orient the mitotic spindle via the dynein-associated protein Mud ^65^. Computational analysis showed that tricellular junction bipolarity forms as the dividing cell elongates, and this geometric orientation facilitates cell mechanical strain-induced polarization during cytokinesis ( Figure 3B). In general, intercellular junctions are important for mechanotransduction between dividing cells and neighboring cells and help establish the orientation of the dividing cells. The coordination of these dynamics of dividing cells with the neighboring cells helps ensure the integrity and fluidity of the forming tissues.

## Summary

Cytokinesis across phyla is a fascinating, highly orchestrated process that requires the interactions of apparently diverse molecular and mechanical machinery and properties. From intracellular coordination with mitosis and complex mechanical networks acting across length- and time-scales to the coordination with neighboring cells, cytokinesis remains a powerful instructive model process for cell and tissue morphogenesis. Furthermore, cytokinesis exemplifies the beauty of robustness by utilizing highly adaptive and mechanoresponsive machinery to ensure high-fidelity cell division, helping to preserve genomic integrity.

Many fundamental questions remain to be fully addressed while new exciting ones continue to arise. For example, how are constriction forces generated and robustness of the contractile machinery achieved across different species? What are the relative contributions of ESCRTIII organized into filaments versus those promoting vesicle generation? How does nuclease resolve chromatin bridges? How does the intercellular force transmission between dividing and neighboring cells contribute to tissue shape and fidelity? Answering these questions will improve our understanding of cytokinesis and will open up many new exciting areas of inquiry about cell and tissue morphogenesis more broadly.
